# Sexual specialization in phenology in dioecious *Ficus benguetensis* and its consequences for the mutualism

**DOI:** 10.1186/s40529-015-0113-7

**Published:** 2015-11-30

**Authors:** Shang-Yang Lin, Lien-Siang Chou, Bruno Di Giusto, Anthony Bain

**Affiliations:** 1 Institute of Ecology and Evolutionary Biology, College of Life Science, National Taiwan University, Rm. 635, Life Science Building, #1, Sec. 4, Roosevelt Rd., Taipei, 10617 Taiwan; 2 Journalism and Mass Communication Department, International College, Ming Chuan University, 250 Zhong Shan N. Rd., Taipei 111, Taiwan

**Keywords:** Dioecy, Evolutionary conflict, *Ficus benguetensis*, Phenology, Taiwan

## Abstract

**Background:**

Timing of reproductive events has become central in ecological studies linking success in pollination and seed dispersion to optimizing the probability and periods of encounters with pollinators or dispersers. Obligate plant–insect interactions, especially *Ficus*–fig wasp mutualisms, offer striking examples of fine-tuned encounter optimization as biological cycles between mutualistic partners are deeply dependent on each other and intertwined over generations. Despite fig flowering phenology being crucial in maintaining *Ficus*–fig wasp mutualisms, until now, the forces of selection shaping the phenological evolution of dioecious fig trees have received little attention. By conducting a 2-year survey of a population of *Ficus benguetensis* in Northern Taiwan, we assessed whether environmental factors or other selective pressures shape the phenology of male and female fig trees.

**Results:**

Constraints by mutualistic pollinating wasps and seed dispersers, rather than climatic factors, appeared to mainly shape fig phenology and allometry in *F. benguetensis*. We identified a new sexual specialization in dioecious fig trees: the position of fig production. We propose that the continuous male fig production on tree trunks can enhance the survival of pollinating fig wasps through faster localization of receptive figs while reducing the mutualistic conflict between the fig and its obligate pollinators. By contrast, in female trees, fig production is massive in summer, located on the twigs of the foliar crown and seem more related to seed dispersal and germination.

**Conclusions:**

Identifying variations in the allometry and phenology of dioecious figs provide valuable insights into how monoecious and dioecious species resolve mutualism conflicts and into the emergence of dioecy in fig trees.

**Electronic supplementary material:**

The online version of this article (doi:10.1186/s40529-015-0113-7) contains supplementary material, which is available to authorized users.

## Background

Knowing the phenology of flowering and fruiting is essential for understanding the ecology and evolution of a plant (Forrest and Miller-Rushing 2010). Moreover, phenological shifts in flowering/fruiting periods may severely affect the associated community of a plant (Miller-Rushing et al. 2010), particularly for interacting species such as obligate mutualistic partners. The failure of mutualistic partners to meet at the appropriate time for pollination or seed dispersal can disrupt biological cycles and cause the extinction of both partners. However, understanding the mechanisms underlying mutualistic interactions requires obtaining precise knowledge on phenology. To assess the role of phenology in the maintenance and evolution of mutualisms, fig trees (*Ficus*, Moraceae) are appropriate models because they have various species, ecologies, reproductive strategies, and phenological cycles (Harrison et al. 2012), and are all involved in obligate pollination mutualism. Generally, each *Ficus* species is pollinated by a sole species of pollinating fig wasp (Hymenoptera: Chalcidoidea: Agaonidae) that breed and develop only inside the female flowers of their host.

Approximately half of *Ficus* species are functionally dioecious (Berg 1989) and present physiological (Dumont et al. 2004) as well as phenological (Valdeyron and Lloyd 1979) adaptations that fulfil reproductive functions. Male fig trees bear figs that produce pollen and pollen vectors. The short-styled ovaries of such figs provide suitable oviposition and larval development sites for pollinators (Kjellberg et al. 2005). Such figs ensure the viability of fig wasps, but produce no seeds (Kjellberg et al. 1987). Male trees continuously produce figs (Dunn et al. 2008); this adaptation was suggested to fit their short-lived pollinators. By contrast, female trees produce seeds (Berg 1989) (female figs produce no pollen) and maintain the *Ficus* biological cycle. Pollinating fig wasps fertilize the flowers inside female figs and ensure seed generation, but fail to produce any offspring because long-styled ovaries form a mechanical barrier to prevent oviposition (Kjellberg et al. 1987). Thus, female trees are a population sink for wasps. This conflict between wasp interest (avoiding female figs) and fig tree interests (pollination of female figs and seed production) is considered solved through phenology (Anstett et al. 1997). For instance, some dioecious species exhibit intrasexual synchronized flowering phenologies (Kjellberg et al. 1987; Patel 1996), where trees from one sex crop after the other. In such systems, pollinators have no choice but to enter the receptive fig of whichever sex is available. Thus, alternated phenologies stabilize the mutualism. In other dioecious species, male and female trees simultaneously flower, thus offering a choice to pollinating wasps. However, two studies (Soler et al. 2011, 2012) have suggested that combined phenological synchrony and intersexual mimicry (resembling scents in receptive figs of both sexes) can reduce the ability of fig wasps to actively choose male figs. In addition, these studies have supported the concept of alternative or additional adaptations to phenology allowing fig trees to “slave” their pollinators into pollinating female figs.

Fig flowering phenology is considered crucial in perpetuating the *Ficus*–fig wasp mutualisms. The deep embedment between the biological cycles of the insect and fig tree strongly relies on synchronized phenologies. For example, some tropical dioecious and most monoecious *Ficus* species present a synchronous intratree flowering combined with a high intertree asynchrony. This differential phenology ensures the permanent availability of receptive figs and the survival of the pollen carriers (Patel 1996; Bronstein et al. 1990). However, until now, the forces of selection that shape the phenological evolution of dioecious fig trees have been overlooked (Anstett et al. 1997; Patel and McKey 1998). While plant phenology has long been considered driven by climatic factors (Körner and Basler 2010), recent studies have emphasized taking account of additional features such as phylogeny (Davis et al. 2010), genetic diversity (Yang et al. 2014), ecology (Forrest and Miller-Rushing 2010), and physiology (Keller et al. 2011) to disentangle climatic and biological selective pressures. In the dioecious fig–fig wasp interactions, a strong sexual selection is expected in life history traits that facilitate, synchronize, and coordinate the interaction between different partners (i.e., male and female trees, fig trees and fig wasps, and fig trees and seed dispersers). We predict that male trees will exhibit a continuous production of figs over a year and locate the fig production over a tree to maximize the probability and speed of localization, and hence, the survival of their obligate pollinators. Conversely, we predict that female trees will present a production limited in time to the optimal season for seed germination and seedling growth as well as locate their fig production for effective fig pollination and dispersal.

We investigated the factors affecting the phenology of *Ficus benguetensis*, a dioecious species involved in a constrained mutualism. By conducting a 2-year survey of a fig population in Northern Taiwan, we assessed whether the plant phenology in male and female trees is shaped by environmental factors or other selective pressures. We hypothesized that sexual specialization in different reproductive functions of male and female trees should be reflected not only in the fig phenology but also in the fig distribution over an individual tree. Identification of such adaptations could provide invaluable cues for understanding the role of phenology and dioecy in the ecology and evolution of mutualisms.

## Methods

### Study species

*Ficus benguetensis* Merr., a functionally dioecious fig species belonging to the subgenus *Sycomorus* and the section *Sycocarpus*, is pollinated by *Ceratosolen wui* (Chen and Chou 1997). This fig species is distributed in the Philippines, Taiwan, and the Japanese Ryukyu Islands (Berg and Corner 2005). It is a small to medium-sized tree (4–10 m height) growing at low altitudes, particularly in valleys with high humidity. Figs grow on branches or directly on the trunk.

**Fig. 1:**
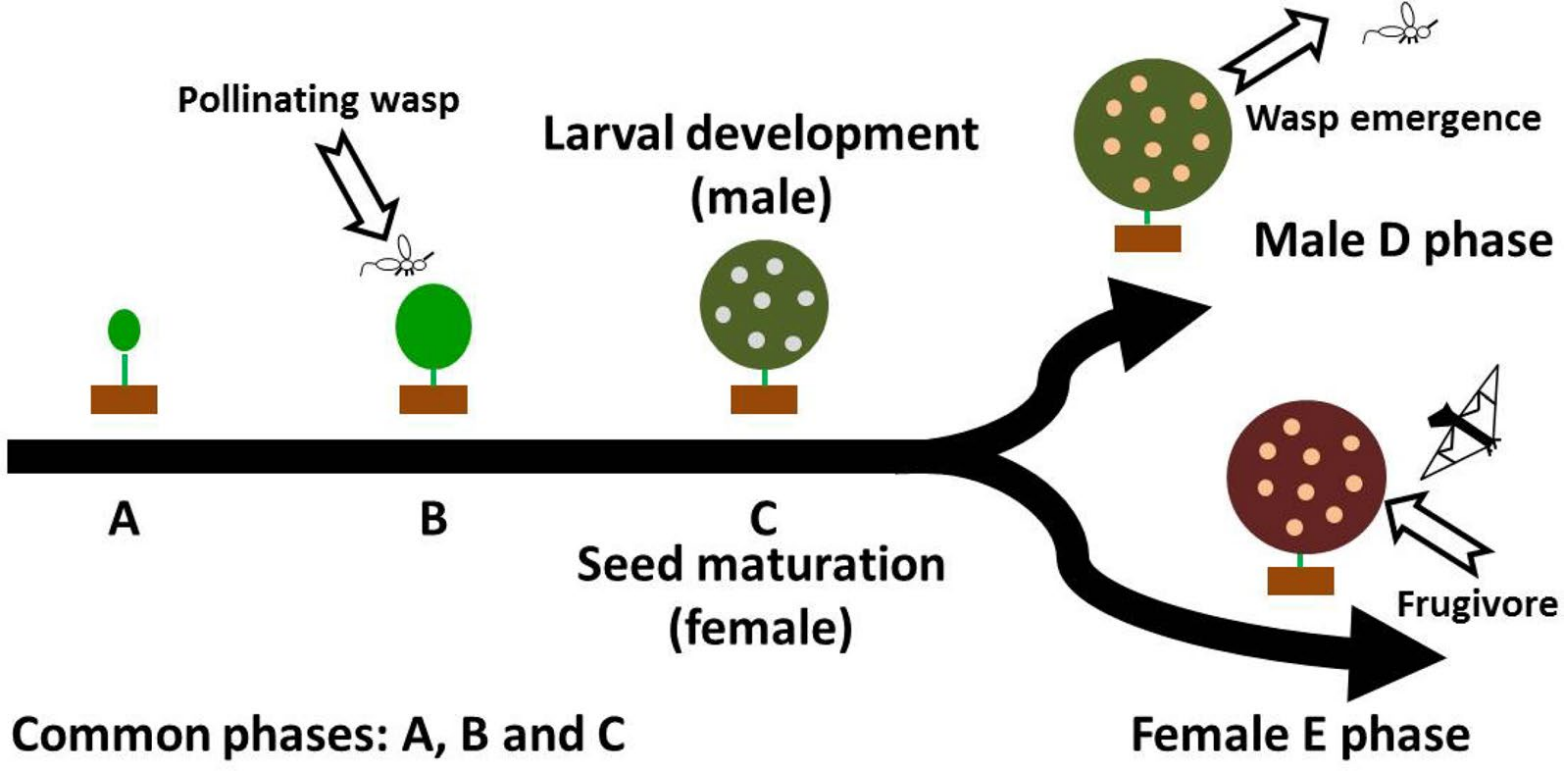
Developmental progress of a dioecious *Ficus*. Prereceptive stage (*A phase*) begins with the appearance of the fig buds. When figs are ready to be pollinated, the receptive stage (*B phase*) begins. From the outside, the bracts slightly open to permit the mutualistic wasp to enter. After pollination, the bracts close and the interfloral stage begins (*C phase*). At this stage, pollinating wasp larvae develop exclusively within the ovaries of male figs, which are transformed into galls. The development of male figs and wasp larvae finishes with the wasp-releasing stage (*D phase*) when the stamens are mature and adult wasps exit their natal galls. In female trees, the final stage is the ripe stage (*E phase*), coinciding with seed maturation and frugivore attraction

According to the terminology for dioecious figs (Galil and Eisikowitch 1968), including the five developmental stages of figs, *F. benguetensis* exhibits three common stages, with the final stage differing in each sex (Fig. 1).

### Phenological census

To investigate the phenology of *F. benguetensis* without anthropic interferences, a natural habitat was selected near Xindian District, New Taipei City, Taiwan (E121°33′55.44; N24°53′49.56).

Seventeen male trees and seven female trees were surveyed weekly, and fig abundance and developmental stages were recorded from March 2011 to March 2013 (25 months). After 10 months of monitoring, noticeable differences in production were observed regarding fig positions on a tree. In addition to the trunk area (between 120 and 150 cm high; Additional file 1: Figure S1), two other areas (30 cm long), the lower part of the first branches and the terminal twigs, were monitored for the second part of the survey. Fourteen male trees and five female trees were then assessed for 15 months from January 2012 to March 2013; three individuals were excluded because their branches and twigs were inaccessible.

Weather data from the Quchi meteorological station of the Taiwan Central Weather Bureau, located approximately 3 km from the study field, were also recorded weekly. During the census period, the weekly mean temperature was 21.3 °C, ranging 12.7–30.0 °C, whereas the annual cumulated precipitation was 3992.25 mm (Additional file 1: Table S1).

### Data analysis

To evaluate the synchronization of figs within a tree, the evenness index, modified from Smith and Bronstein (1996) was used. This modification considers the difference in the duration of each developmental phase of a fig for each sex, a parameter critical to calculate the probability of their occurrence. The fig evenness within a tree, E, for each sex was calculated as follows: E = ΣPi × ln [(Pi/Di) + 1], where i represents each developmental stage, Pi represents the proportion of figs at stage i, and Di represents the expected duration proportion of each developmental phase. Values were then calibrated to range from 0 (total asynchrony) to 1 (total synchrony). An average duration for each developmental stage was calculated based on the measures obtained during our 2-year survey.

To explain the observed patterns of fig production in *F. benguetensis*, an estimator of the total yield of a tree, Y, was designed. Fig abundance was first measured based on the samplings of the three areas of production of a tree: trunk, branch, and twigs. Subsequently, the total yield (Yi) was estimated as follows: Yi = Ti + 2.5·Bi + 150 Wi, where Ti represents the number of figs produced by the sampled area on the trunk of the tree i, Bi represents the number of figs on its sampled branch, and Wi represents the number of figs on its sampled twigs. The coefficients were estimated according to the average number of branches observed per tree (i.e., 2.5), and the average number of twigs observed per tree (i.e., 150).

Statistical analyses were performed using SYSTAT v.12. The Mann–Whitney U test and Kruskal–Wallis test were used to compare pairs of data sets and multiple-factor data sets. Moreover we use simple regressions to verify whether dependent fig phenological variables were explained with independent climatic variables (Pereira et al. 2007). Since phenology data sets generally exhibit temporal autocorrelation (i.e., non-independent error variance) and thus break the assumptions of serial independence required for most inference tests (Pyper and Peterman 1998). Durbin–Watson test was employed to check for temporal autocorrelation (Chatterjee and Price 1991). Because our phenology data is structured in time series, we used ordinary least squares (OLS) models with auto-correlated errors (Venables and Ripley 1999). These regression analyses were conducted using the Proc AUTOREG procedure of SAS 9 (SAS Institute Inc. 2002).

## Results

### Crop number, fig abundance, and evenness

**Fig. 2:**
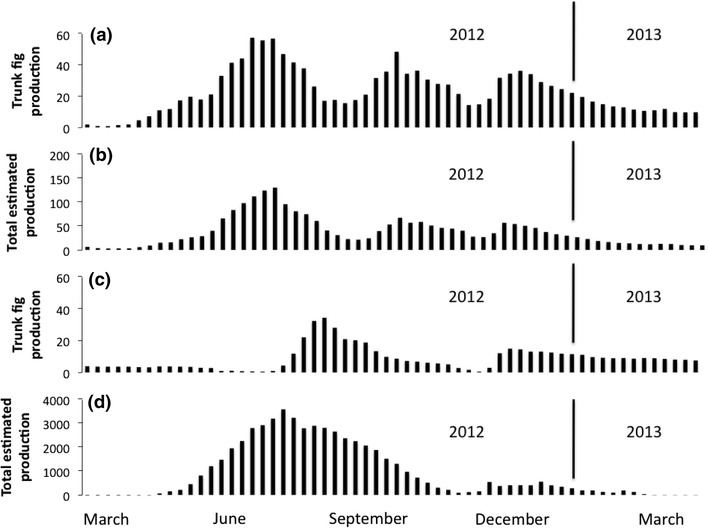
The fig production of male trees (**a**, **b**) and female trees (**c**, **d**) during March 2012–March 2013. The *graphs* **a** and **c** represent the trunk production and the *graphs* **b** and **d** represent the total estimated production

The phenological pattern in fig production of *F. benguetensis* showed clear differences between both sexes. First, male trees produced significantly more crops (2.08 ± 1.13) than did female trees (1.36 ± 0.90) (Table 1). Furthermore, male trees began producing figs earlier in spring and grew figs continuously, except for a short gap in winter. By contrast, female trees produced fewer crops within a year, most of them being restricted to summer (Fig. 2). Second, the average fig abundance on the trunk of male trees (24.60 ± 16.00) was significantly greater than that on the trunk of female trees (9.74 ± 9.21) for the entire survey period (Table 1).

**Table 1:** Phenological characteristics of *Ficus benguetensis*

	Crop number	Crop onset (days)	Fig abundance	Evenness
Male	2.08 ± 0.27 (17 trees)	49.50 ± 6.10 (28 crops)	24.60 ± 1.58 (102 surveys)	0.29 ± 0.01 (916 observations)
Female	1.36 ± 0.34 (7 trees)	78.45 ± 16.19 (11 crops)	9.74 ± 0.91 (102 surveys)	0.21 ± 0.01 (364 observations)
Mann–Whitney test	U = 388*	NS	U = 8224**	U = 9001**

The mean number ± SE (sample size)

** *P* < 0.01, * *P* < 0.05

Trees from both sexes had low values of average evenness (<0.3), but higher asynchrony was exhibited by female trees than by male trees (Table 1). The extent of the evenness values (0.148–0.773 and 0.085–0.657 for male and female trees, respectively) was driven by major variations in the estimated duration of their different developmental phases. Moreover, evenness was similar in the growth areas of male trees: 0.31 ± 0.29 for the trunks and 0.27 ± 0.27 for the branches [Mann–Whitney test: non-significant (NS)], but not in the growth areas of the female trees: 0.26 ± 0.29 on the trunk, 0.17 ± 0.23 on the branches, and 0.16 ± 0.20 on the twigs (Kruskal–Wallis test statistic: 15.9, df = 2, *P* < 0.001). The Mann–Whitney tests for the three fig-bearing positions showed that the evenness on the twigs was significantly lower than that on the trunk and branches (twig/branch: *P* < 0.01; twig/trunk: *P* < 0.001; trunk/branch: NS; Additional file 1: Figure S2).

### Crop allometry

In comparing the production of the three fig-bearing positions (trunk, branch, and twig), we discovered significant differences between the sexes. Because no male trees produced figs on their twigs, the distribution of fig abundance was highly skewed among the fig positions in both sexes. For male trees, figs were more abundant on trunks than on branches: 24.60 ± 16.00 figs and 6.19 ± 6.99 figs, respectively (Mann–Whitney test: *P* < 0.001). By contrast, female trees produced 9.74 ± 9.21, 6.00 ± 7.25, and 2.96 ± 2.41 figs on trunks, twigs, and branches, respectively (Kruskal–Wallis test: 26.994, df = 2, *P* < 0.001).

**Fig. 3:**
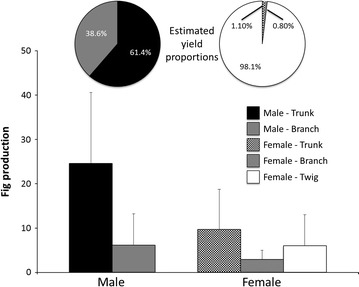
Fig production and yield by locations in 2012. The *upper* pie charts show the proportions of estimated fig yields from different tree positions and sexes. The *lower column* charts show the mean and standard variation of the fig numbers counted on sampled tree positions. Male trees produced no figs on twigs. Fig production on the trunk of male trees was significantly higher than that on the trunk of female trees. Fig production by male and female trees was significantly different between positions (Mann–Whitney U test for males: *P* < 0.001 and Kruskal–Wallis test for females: *P* < 0.05)

Regarding yields per entire tree, the average estimated fig yield for female trees, Y_F_, indicated that 98.13, 1.06, and 0.08 % of the fig production was located on the twigs, trunks, and branches, respectively (Fig. 3). Conversely, the average estimated fig yield for male trees, Y_M_, indicated that most of the fig production was from the trunks (61.38 %), with no production from the twigs. Thus, during the second year, the estimated fig production by female trees was considerably higher than that by male trees (916.21 ± 1096.41 figs and 38.55 ± 30.71 figs, respectively; Mann–Whitney test: *P* < 0.001; Fig. 2).

### Pollination windows

The overlapping of receptive (B) and emerging (D) fig phases allows three possible destinations for a newly emerged fig wasp from D-phase figs on male trees: The insects can enter a B-phase fig on (1) the same tree, (2) a different male tree, or (3) a female tree. During our survey period, B–D events occurred on the same tree and day (self B–D) 18 times in 102 surveys and in only four male trees (23.5 % of the male trees). Among those 18 events, 13 occurred on the trunk (Table 2). No self B–D event occurred on the branches. At the population level, B–D events were slightly more abundant because the sample size was also greater. The matching between male D-phase figs and female B-phase figs (female B–D) occurred 32 times in 102 surveys, and 45 times among different male trees (male B–D) (Table 2).

**Table 2:** Proportion of B phase–D phase events within male individual (self B–D); between males (male B–D); and between male and female (female B–D)

	Self B–D	Male B–D	Female B–D
Trunk only			
Number of surveys	10	42	28
Total number B and D phases figs observations	418	418	237
All locations			
Number of surveys	12	45	32
Total number B and D phases figs observations	539	539	344

The total number of observations is 4701 (survey number × tree number × tree position) while the total number B and D observations calculated by adding up the number of observations of B and D figs

### Climatic factors

The number of figs in phase A (beginning of the crops) and fig abundance showed time series autocorrelation in both male (Durbin-Watson D Statistic: 0.419, p < 0.001 and 0.343, p < 0.001, respectively) and female (Durbin-Watson D Statistic: 0.461, p < 0.001 and 0.317, p < 0.001, respectively). None of the phenological variables (beginning of the crop and fig abundance in both sexes) did not display any significant correlations with climatic variables (temperature and rainfall).

## Discussion

Our study shows that the phenology of fig production in *F. benguetensis* is the product of biological processes. The variation observed in fig production over time could not be attributed to temperature or rainfalls, whereas male and female trees growing in the same environment exhibited distinct phenologies.

Because this mutualism is obligate and because dioecious individuals serve different reproductive functions, distinct spatial and temporal adaptations were selected to enhance the effectiveness of each function. Male *F. benguetensis* figs were continuously available at the receptive stage for the pollinating fig wasps through various mechanisms. Male trees presented both a greater number of major crops per year and a small production of figs between with major crops, which resulted in an overall continuous fig production. All male figs were located only on the trunk and the lower parts of main branches, and showed a high level of intratree and intertree fig asynchrony; all of which are traits inclined to enhancing the survival of pollinating fig wasps. By contrast, female trees produced fewer crops, all during the summer, but exhibited a mass production of highly asynchronous figs located on the twigs. We argue that understanding how dioecious fig trees reduce the evolutionary conflict with the pollinating fig wasps cannot be deduced solely from phenological surveys. Fig distribution over a tree and the tree sex ratio are critical for understanding the maintenance and evolution of dioecious fig trees and their pollinators.

Identifying the intersexual differences in the allometry of fig production is the most striking result of this study. Quantitative surveys of the reproductive phenology of dioecious fig species have shown numerous sex differences in the timing of fig production, the size of the figs, and the duration of fig developmental stages (Patel and McKey 1998; Chou and Yeh 1995; Harrison and Yamamura 2003; Bain et al. 2014). While a few pluriannual and quantitative surveys have already been carried out over 2 (Patel 1996) and 3 years (Corlett 1993), our study is the first to report differences in both fig phenology and distribution over various positions on a single tree. Male trees bore two-thirds of their figs on their trunks and not on their twigs, whereas nearly all of the fig production of female trees was located on the twigs of the foliar crown. We consider the adaptations of *F. benguetensis* because of sexual specialization and their evolutionary implications as follows.

In dioecious species, the necessity to maintain the pollinator biological cycle (Kjellberg et al. 1987) with a constant supply of figs shapes the spatial and temporal distributions of the figs over an individual male tree. The general pattern of cauliflorous male fig production in *F. benguetensis* (spring and autumn production peaks and higher male fig production) is consistent with that in other dioecious *Ficus* in Taiwan (Bain et al. 2014) or the Asian continent (Yu et al. 2006). Our analysis reveals several mechanisms maximizing the chances of survival of pollinating wasps despite their estimated lifespan of 12 h to 3 days (Dunn et al. 2008). As predicted, male trees optimize the survival of their obligate pollinators by maximizing the probability of fig localization. Investing most of the male fig production in cauliflory, a common strategy for tropical fig species (Berg and Corner 2005), allows retrieving a part of the nutrients invested in the fruits (Harrison and Yamamura 2003) and offers various selective advantages for male fig trees and insects. Heavy rains often result in most of the produced figs falling on the ground or remaining unpollinated (Lin S-Y, National Taiwan University, Taiwan, unpubl. res.). The foliar crown of a fig tree may protect its cauliflorous crops by shielding them from violent rainstorms. Additionally, the pollinators of dioecious fig trees seem to favour dispersal at short distances and through the understorey (Harrison and Rasplus 2006). Because pollinating fig wasps are sensitive to desiccation (Dunn et al. 2008) flying under the canopy could ensures higher survival rates for the following reasons. First, the understorey environment is more humid and has reduced thermal amplitude (Lebrija-trejos et al. 2011). Second, the cauliflorous figs of male trees are most abundant in the understorey; hence, they are more likely to be discovered by pollinating fig wasps. Third, because male trees are relatively more abundant than female trees, the flying distance between two male figs is often shorter than between a male fig and a female fig. Our sampling biased toward male trees could simply reflect a general trend observed in *F. benguetensis* and other dioecious *Ficus* trees in Taiwan: 352 observed *F. benguetensis* on Taiwan Island, 70 males, 47 females and 235 unidentified individuals (Bain A, National Taiwan University, Taiwan, unpubl. res.). Finally, intratree and intertree asynchronies maximize an extensive availability of receptive figs for pollinators. The relationship between intratree and intertree asynchronies for various densities of *F. benguetensis* populations or other fig species should be explored. We hypothesize both a higher intratree asynchrony [fig trees simultaneously presenting the fig receptive stages (B) and wasp-releasing stages (D)] and a male-biased sex ratio in pioneer *Ficus* populations (isolated trees or sparsely distributed fig populations) that perpetuate pollinator populations (Alvarez et al. 2010).

Our study also revealed specific adaptations in female trees. Although present, cauliflory represented only 1 % of the estimated female fig production. This result was expected because seeds from fallen figs are unlikely to germinate if they are too close to their genitor. The change in resource allocation towards the twigs seems more linked to seed dispersal. Similar to other species of the *Sycomorus* subgenus, *F. benguetensis* is dispersed by fruit bats (Harrison et al. 2012). In contrast to *F. racemosa* and *F. variegata* (Patel et al. 1995), two tall and cauliflorous tree species dispersed by bats, *F. benguetensis* is a medium-sized tree, with its foliar crown often reaching the canopy in Taiwan forests. Producing female figs on the terminal twigs can facilitate the access and consumption of ripened figs by bats, and then increase the chances of effective seed dispersal. Additionally, the highest intratree asynchrony of female figs growing on twigs can further increase dispersal by ensuring repeated visits by frugivores. This extreme asynchrony seems directly linked to longer fig developmental stages. We estimated the total duration of development at 121 days for female figs and 71 days for male figs. Such extended prereceptive and maturation phases of the female figs are likely due to a greater number of ovaries in female figs and the necessity to be fleshier to attract seed dispersers (Patel and McKey 1998). Furthermore, a high intratree fig asynchrony ensures that female trees experience an extended and greater variety of environments for seed germination (Patel et al. 1993).

Our data support the recommendation by Harrison et al. to reassess the assumption that fig cauliflory is an evolutionary adaptation to bat dispersal (Harrison et al. 2012). Intersexual differences in allometry indicate that cauliflory in *F. benguetensis* is likely an evolutionary response that enhances pollination, whereas ramiflory is apparently an adaptation to seed dispersion by fruit bats. However, despite such differences between male and female figs, it remains unclear why the pollinator *Ceratosolen wui* did not “learn” to avoid female figs that entail such a fitness loss for the insect. Two main hypotheses with a few subhypotheses assuming intersexual chemical mimicry (Patel et al. 1995) may explain this absence of pollinator discrimination in synchronous male and female trees. First, the “no preference” hypothesis states that pollinating wasps are simply unable to distinguish between male and female figs. This inability could be due to vicarious selection leading to complete intersexual chemical mimicry (Grafen and Godfray 1991) or to the absence of selection by fig wasps to favour a specific sex (Anstett et al. 1997). An intersexual comparison of the composition and quantities of the volatiles attracting pollinating wasps, followed by insect bioassays, is necessary to choose between these two competing subhypotheses. Second, the “limited partial preference” hypothesis, which is based on imperfect sex mimicry, states that pollinators might develop a partial preference for male figs. However, this partial preference is a frequency-dependent mechanism susceptible to disappearing in a male-dominated environment (Getty 1985), particularly if males present individual scents that vary excessively. Such variability, a common trait in *Ficus* scents (Soler et al. 2012), can increase the risk of a tree not being recognized as male, thus increasing the risk of a pollinator never entering a male fig (Patel et al. 1995). Finally, another subhypothesis of limited partial preference is called “selection to rush” (Patel et al. 1995). Although pollinating wasps may discriminate between male and female figs, their lifespan is a constraint and they cannot afford the reproductive cost incurred by the delay of choosing between male and female figs. Because wasps face limited availability and high competition for male receptive figs, wasps that rush into any receptive fig, whether male or female, are likely to be strongly selected. Therefore, we hypothesize that the combination of the short lifespan and the dispersion range of the pollinator could counter-select any partial preference of the insect. Moreover, such a system could produce an overload of pollen vectors, thereby triggering a scramble competition for a few receptive male figs. Pollinating wasps rush to enter the closest available receptive fig (likely a male fig), and then enter other figs at increased distances. Female figs available on the canopy in an extremely patchy distribution are pollinated because of the number of pollinators and their physical position acting as a pollinator net for the pollinating wasps that escaped the understorey. Further research is necessary to determine the sex ratio in various populations of *F. benguetensis* and to assess the lifespan and capacity of the dispersion of *Ceratosolen wui*.

Dioecy in *Ficus* is considered a characteristic derived relative to monoecy (Berg 1989). Although dioecy was proposed to be particularly adapted to seasonal climate (Kjellberg and Maurice 1989), alternative hypotheses, such as parasitic pressure (Kerdelhué and Rasplus 1996) or ant predation (Harrison and Yamamura 2003), have been subsequently developed to explain the appearance of dioecy in *Ficus*. A study on *F. exasperata* and *F. hispida* observed strong seasonal patterns in fig production (Patel and McKey 1998); however, in the present study, we observed no such strong seasonal patterns in *F. benguetensis*, except for the patterns of the estimated yield of female figs growing on twigs. Our results do not support the hypothesis that the appearance of dioecy could result from a differential allocation to reproductive functions among seasons (Kjellberg and Maurice 1989). However, our observations agree with the concept proposed by Patel and McKey (1998), that extreme sexual specialization can be an adaptive response to unstable trade-offs in the reproductive traits of monoecious figs. We hypothesize that, ancestrally, male and female trees could produce figs similarly on both trunk and twigs, and that male and female figs may be indistinguishable for the pollinator. The superior survival of pollinating wasps that enter cauliflorous figs and the cost of producing less effective figs on twigs would have resulted in a directional selection in male trees toward exclusive cauliflory. Conversely, female trees bearing major trunk crops could drastically reduce local wasp populations and should be counter-selected in the long term. In addition, the absence of male figs on twigs is advantageous because it prevents the selection of wasps preferring the canopy environment. Finally, differences in sex ratios could be the response of fig trees to the development of a partial preference for male figs by the insects. Further study on related and unrelated dioecious *Ficus* growing in an aseasonal environment could shed light on other proximate factors that led to dioecy in fig trees.

In summary, sexual specialization and biological requirements of the agaonid wasp *C. wui*, but not climatic factors, appear to mainly shape the phenology and allometry in the simultaneously flowering dioecious *F. benguetensis*. We identified a new sexual difference in dioecious fig trees: the position where the figs are produced. If such an evolutionary response provides a selective advantage to the maintenance of the pollinator population and to the decrease in the negative impact of the female trees, other dioecious *Ficus* could have taken the same evolutionary route. The survey of intraspecific and interspecific variations in the allometryand phenology of dioecious figs could provide valuable insights into how monoecious and dioecious species resolve mutualism conflicts and on the emergence of dioecy in fig trees.

## Additional file

**Additional file 1.** The supplementary materials display additional methods descriptions such as meteorological data and sampling methods as well as detailed results on average evenness.
