# Supplementary material for: Sexual specialization in phenology in dioecious Ficus benguetensis and its consequences for the mutualism
Source: Bot Stud. 2015 Nov 30;56:32. doi: 10.1186/s40529-015-0113-7 (PMC5432922; doi:10.1186/s40529-015-0113-7)
Supplement: Supplementary file 1 — Additional file 1. The supplementary materials display additional methods descriptions such as meteorological data and sampling methods as well as detailed results on average evenness. [file 40529_2015_113_MOESM1_ESM.docx]

**Supplementary materials**

**Table S1**. Weekly temperatures and precipitations during the survey period. The mean values and the standard deviation are displayed.

|  | Temperatures (°C) | Precipitations (mm) |
| --- | --- | --- |
| 2011 |  |  |
| Spring | 18.21 ± 3.60 | 55.58 ± 71,62 |
| Summer | 25.90 ± 1.24 | 78.29 ± 75.78 |
| Autumn | 21.75 ± 2.37 | 61.35 ± 68.23 |
| Winter | 15.69 ± 1.56 | 75.12 ± 45.58 |
| 2012 |  |  |
| Spring | 21.87 ± 2.94 | 68.58 ± 49.88 |
| Summer | 27.92 ± 1.30 | 145.3 ± 205.0 |
| Autumn | 22.78 ± 2.94 | 91.96 ± 175.7 |
| Winter | 16.47 ± 1.58 | 36.27 ± 35.91 |


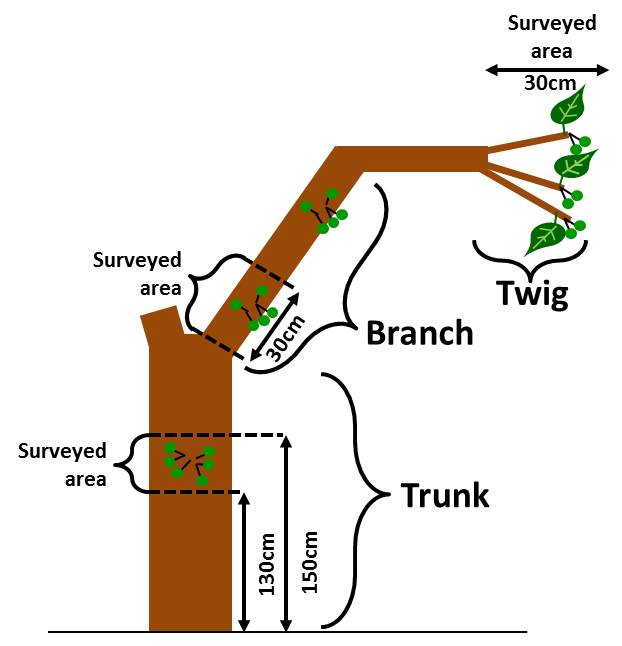


**Figure S1.** Schematic of the 3 monitored locations of the phenological census.


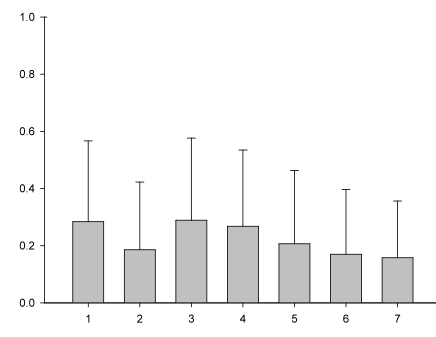


**Figure S2.** Average evenness (and standard deviation) of fig production in various positions on male and female trees: 1. Total male evenness; 2. Total female evenness; 3. Male trunk evenness; 4. Male branch evenness; 5. Female trunk evenness; 6. Female branch evenness; 7. Female twig evenness.
